# Atomic Step Formation on Sapphire Surface in Ultra-precision Manufacturing

**DOI:** 10.1038/srep29964

**Published:** 2016-07-22

**Authors:** Rongrong Wang, Dan Guo, Guoxin Xie, Guoshun Pan

**Affiliations:** 1 State Key Laboratory of Tribology, Tsinghua University, Beijing, 100084, China; 2 Guangdong Provincial Key Laboratory of Optomechatronics, Shenzhen, 518057, China

## Abstract

Surfaces with controlled atomic step structures as substrates are highly relevant to desirable performances of materials grown on them, such as light emitting diode (LED) epitaxial layers, nanotubes and nanoribbons. However, very limited attention has been paid to the step formation in manufacturing process. In the present work, investigations have been conducted into this step formation mechanism on the sapphire c (0001) surface by using both experiments and simulations. The step evolutions at different stages in the polishing process were investigated with atomic force microscopy (AFM) and high resolution transmission electron microscopy (HRTEM). The simulation of idealized steps was constructed theoretically on the basis of experimental results. It was found that (1) the subtle atomic structures (e.g., steps with different sawteeth, as well as steps with straight and zigzag edges), (2) the periodicity and (3) the degree of order of the steps were all dependent on surface composition and miscut direction (step edge direction). A comparison between experimental results and idealized step models of different surface compositions has been made. It has been found that the structure on the polished surface was in accordance with some surface compositions (the model of single-atom steps: Al steps or O steps).

The sapphire is often used as a substrate in the integrated circuits industry, optoelectronic industry and for many scientific applications^1^,^2^,^3^,^4^,^5^,^6^,^7^,^8^,^9^,^10^,^11^. For film and epitaxy, the substrate surface properties greatly affect the growing quality^2^,^4^,^5^,^6^,^12^. Thus, a defect-free smooth surface is essential. Several research teams have produced atomically flat surfaces through ultra-precision manufacturing, and the surfaces had globally regular atomic step-and-terrace structures^13^,^14^,^15^,^16^,^17^. On the one hand, this step structure affects the growth quality of the LED epitaxial layer^18^,^19^,^20^. On the other hand, the step structure on the polished surface could be a bridge to solve the dilemma of bringing the large area and the nanostructure together. It could have important implications in nanoengineering and bioengineering areas for fabricating nanostructures in a large area^21^,^22^,^23^,^24^,^25^,^26^. Moreover, step-and-terrace structures have been used as large scale nanopatterned surfaces to grow thin films, nanoribbons and nanotubes^27^,^28^,^29^,^30^,^31^,^32^,^33^,^34^,^35^,^36^. Theoretical and experimental works have been conducted previously on the step-and-terrace structure formed by annealing and hydrogen-etching, etc^15^,^37^,^38^,^39^. Some other works have been focused on the anisotropy of the sapphire and the interface between the sapphire and other material^40^,^41^,^42^. Furthermore, atomic steps have been often merely regarded as one of the criteria for the evaluation of the quality of ultra-precision surface. However, research regarding the step formation after ultra-precision manufacturing had been relatively insufficient. To fill the vacancy, in this paper, the evolution of step formation on sapphire c (0001) surface in ultra-precision manufacturing was investigated with the atomic force microscopy (AFM) and high resolution transmission electron microscopy (HRTEM) measurements. Moreover, step models of different miscuts and terminations were studied theoretically and compared with experimental results.

## Results

### Experiment results

**Figure 1:**
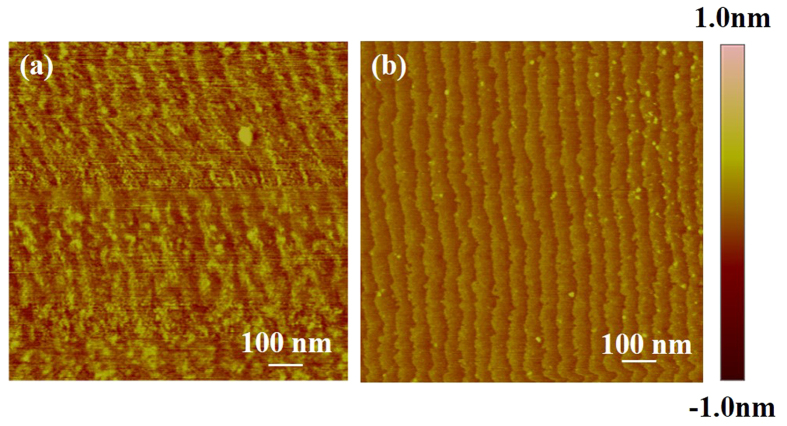
AFM measurements of sapphire wafer after polishing. Surface morphologies evolved from (**a**) blur steps to (**b**) clear steps as the polishing progressed.

**Figure 2:**
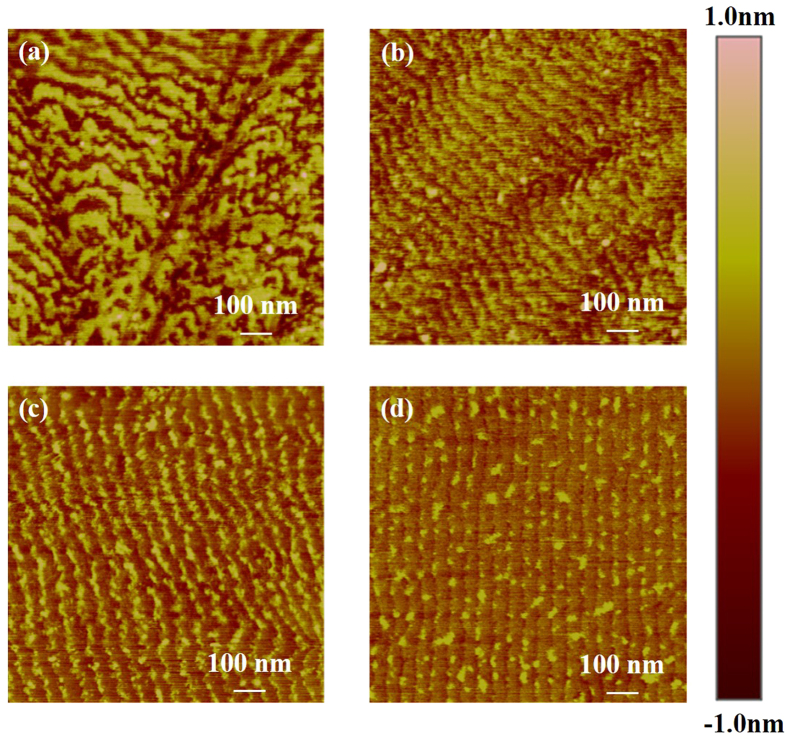
AFM topographies of step evolution on the scratched surface during the polishing process. (**a**) Steps formed on the peaks. (**b**) Surface with large waves of steps. (**c**) Surface with small waves. (**d**) Surface with straight steps as the damage removed by polishing.

From the AFM topography of the sapphire wafer surface after polishing, it could be seen that the step morphology did not manifest itself at the very beginning but became increasingly obvious. Initially, there were no steps on the surface. After polishing for 20 minutes, blur steps emerged on the surface, as shown in Fig. 1(a). After polishing for 60 minutes, the steps became clear as shown in Fig. 1(b) and their morphologies did not change dramatically as the polishing progressed further. For the damage-free region, the surface invariably evolved from blur steps to clear steps. In contrast, the evolutions of step morphologies with damage were quite different. After polishing for 20 minutes, the steps formed first at peaks which were caused by scratches [shown in Fig. 2(a)], which was probably because the energy of the peak region was higher. After polishing for 40 and 60 minutes, the step morphologies evolved into large waves of steps and then to small waves of steps [shown in Fig. 2(b,c)]. Finally, after polishing for 80 minutes, the steps became straight, as shown in Fig. 2(d). Through the polishing process, regions without damage became smoother and showed clear atomic step-and-terrace structures. Equally, regions with damage could also obtain clear and straight atomic steps after the removal of damage by polishing.

**Figure 3:**
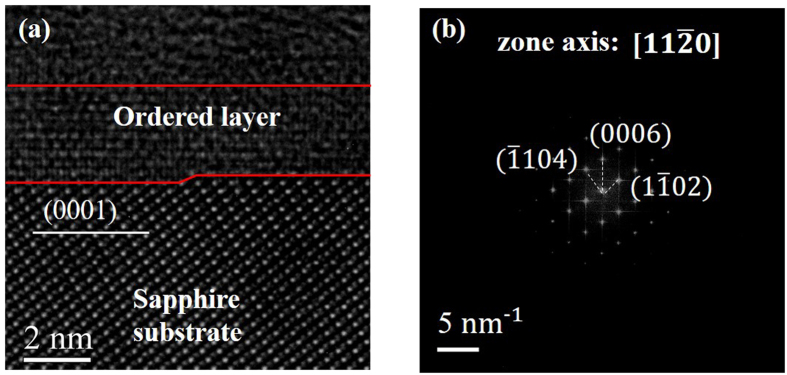
(**a**) HRTEM sectional image of the step morphology. The sapphire wafer was under the protection of noncrystalline carbon (C) and platinum (Pt). The lattices of the sapphire were quite regular. (**b**) The fast Fourier transformation of the HRTEM image. The zone axis was calibrated as 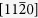, which was the miscut direction (step edge direction).

The HRTEM experiment was conducted to provide more information of the polished surface. From the sectional image of HRTEM [in Fig. 3(a)] and the fast Fourier transformation of the HRTEM image [in Fig. 3(b)], it was calibrated that the zone axis was 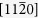. During the HRTEM experiment, the electron beam was under normal incidence in general. The sample was controlled to the greatest extent by FIB to be perpendicular to the step edge and the surface. And the interplanar distance was in good accordance with that of the (0006) plane. The surface of the sapphire wafer used in the experiment was c (0001) ± 0.5°. Hence, the miscut direction (step edge direction) of the wafer was a-axis 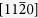. Furthermore, there was an ordered layer above the sapphire surface. It was speculated that the substrate was under the protection of a hydration layer according to the previous research on the removal mechanism of polishing^43^.

### Simulation results

From the AFM experiments, the step height accorded well with the crystal parameters of sapphire. From the HRTEM experiments, the sapphire lattices did not distort. Hence, the study on the step formation with the idealized step-and-terrace structure (which was defined as the step model created by the idealized crystal structure in this simulation) was reliable. Thus, the influences of different miscuts and the compositions were investigated through the statistical data of the step widths of idealized step structures. Moreover, more subtle arrangements of atoms could be observed by building the idealized step structure, which is far more difficult to observe in experiments (see Section S1, S2 in Supplementary Information).

**Figure 4:**
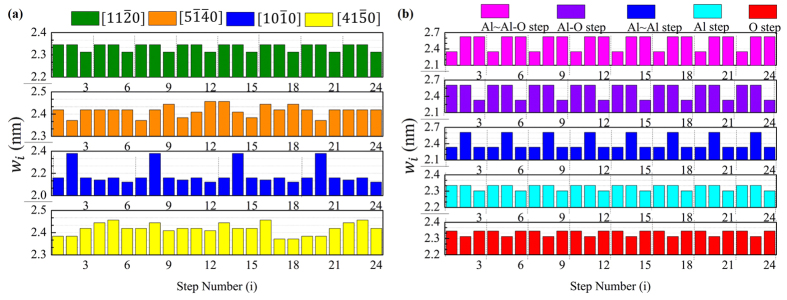
(**a**) Width of each step for the miscut angle (step slope angle) of 5°: different miscut directions (step edge directions); step composition of Al step. (**b**) Width of each step for the miscut angle (step slope angle) of 5°: miscut direction of a-axis; different step compositions.

Statistics of Al step widths were conducted to analyze the miscut influence on the step parameter, taking into consideration that the surface terminated with Al atoms was with the lowest energy as reported theoretically through molecular dynamics^44^. The Al step width of each step (see Section S1.1, *w*_*i*_ was defined as the width of the step i was summarized in Fig. 4(a) when the miscut angle (step slope angle) was 5°. The step widths of 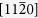 (a-axis) were periodic (with a periodicity of 3 steps), those of the 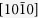 (m-axis) were periodic to a limited extent (with a periodicity of 6 steps), while those of the other two directions had no apparent periodicity. The reason could be attributed to the crystal structure and the atomic distance. There are 6 trilayers in one-unit cell and every two trilayers are chirally symmetrical (see Figure S2). Hence, the periodicity of 3 steps reflected the crystal structure, showing the step structure of a-axis was more regular. If the miscut directions (step edge directions) were sorted from regular step structures to less regular ones, the ranking would be: 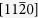 (a-axis) > 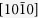 (m-axis) > 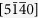 ≈  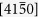, which is in accordance with the analysis of step parameter fluctuations (see Section S3) and the aerial-view surfaces (see Section S2).

As can be seen from the results above, the step structures formed along a-axis were relatively regular. Thus, a-axis was used as the miscut direction (step edge direction) to study the effect of compositions on step structures. It can be seen from Fig. 4(b), all the step widths of different compositions have the periodicity of 3 steps, indicating the composition would not affect the periodicity of the step parameter. However, summarizing the analysis of step parameter fluctuations (see Section S3) and the step sections (see Section S1), the ranking of the compositions sorted from regular step structures to less regular ones would be: Al step ≈ O step > Al~Al step > Al-O step > Al~Al-O step.

### Comparison between the experiments and the simulations

**Figure 5:**
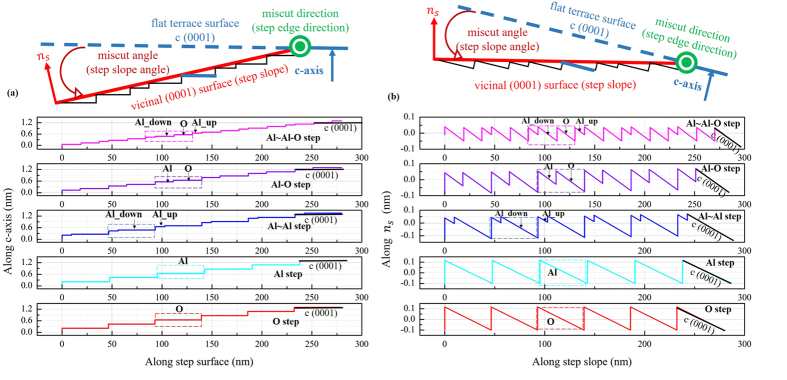
The idealized step structures with the step edge along the a-axis and the miscut angle (step slope angle) of 0.26° were built. (**a**) Step profiles with the horizontal axis along the flat terrace surface and the vertical axis along the c-axis. (**b**) Step profiles with the horizontal axis along the vicinal (0001) surface (step slope) and the vertical axis along *n*_*s*_ (which is normal to step slope).

To better understand the step formation mechanism in polishing, comparisons between the idealized step structures and the experimental results are very helpful. It was calculated from experiments that the miscut direction (step edge direction) was a-axis and the miscut angle (step slope angle) was 0.26° (see Section S4), and the subsequent idealized step structures were built. The c-axis of the idealized step structures was vertical [shown in Fig. 5(a)]. However, for the wafer used in experiments, the normal axis (*n*_*s*_) to the vicinal (0001) surface (step slope) was vertical. Hence, for better comparison with the AFM sectional profiles of the experimentally obtained steps, the profiles of the idealized step structures were converted as shown in Fig. 5(b), where the vertical axis was along *n*_*s*_. It can be seen that the single-atom steps (Al step and O step) are of good self-similarity. One Al~Al-O step had three sawteeth, and one double-layer step (Al-O step and Al~Al step) had two sawteeth. Moreover, the height (herein height means the gap between the highest point and the lowest point in one step) along *n*_*s*_ of Al~Al-O step is about 0.1 nm. The height along *n*_*s*_ of Al-O step is about 0.13 nm. The heights along *n*_*s*_ of Al~Al step, Al step and O step are about 0.2 nm.

**Figure 6:**
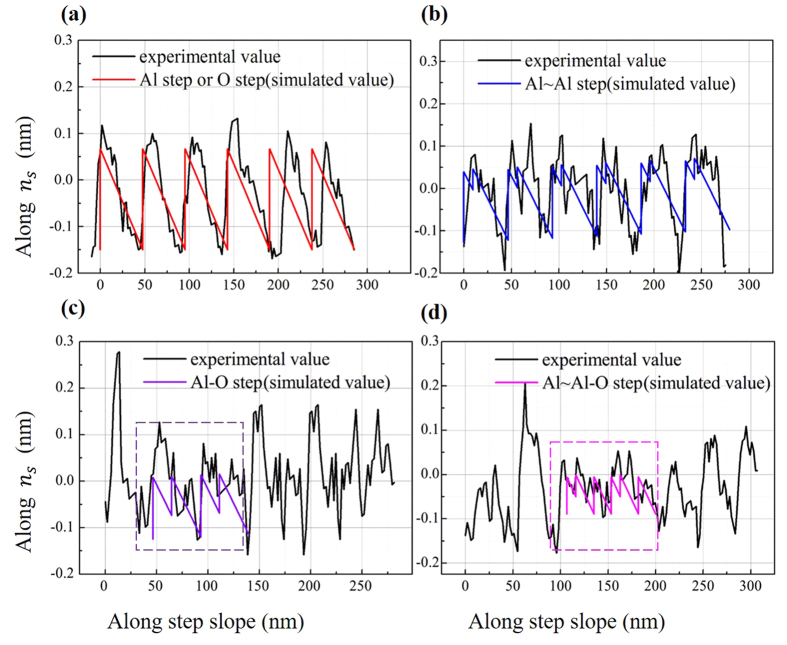
Profiles of the steps in the experiment and the idealized step structures. The experimental profile of (**a**) was extracted from the AFM image of the regions without damage, but the similar profile could be extracted from most AFM images of the regions with damage, too. The profiles of (**b**–**d**) were extracted from AFM images of the regions with damage. More details could be seen in the Section S5. (**a**) Comparison between the experiment and the idealized single-atom steps (Al steps or O steps). (**b**) Comparison between the experiment and the idealized Al~Al steps. (**c**) Comparison between the experiment and the idealized Al-O steps. (**d**) Comparison between the experiment and the idealized Al~Al-O steps.

According to the previous analysis, there are two features to identify different step compositions. One is the number of the sawteeth in one step, and the other is the height along *n*_*s*_ (normal to the step slope). In most areas on the sapphire wafer, the step heights along *n*_*s*_ were about 0.2 nm, and the step profiles in the experiments were in better accordance with Al steps or O steps [see Fig. 6(a)] and in good accordance with Al~Al steps [see Fig. 6(b)]. Therefore, terminations were more likely Al or O, while the possibility of other compositions could not be completely excluded. There were steps with the height along *n*_*s*_ of about 0.12 nm and three sawteeth in very small regions, as exemplified by the profile segment in Fig. 6(d), being in agreement with the Al~Al-O steps to a certain extent. Moreover, there were several steps with the height along *n*_*s*_ of about 0.15 nm and three sawteeth, which roughly accorded with the Al-O steps [see Fig. 6(c)].

## Discussion

The step evolution of different polishing stages on the sapphire c (0001) surface was studied with the AFM results. In the region without damage, the surface evolved from no steps to blur steps and finally to clear steps. In the region with damage, the surface has peaks and valleys, and the steps first appeared at the peaks with the morphology of big waves of steps. As the damage was removed by polishing, the morphology was gradually turned into small waves of steps. Finally, the surface had straight steps and reached a defect-free state. Hence, in general, ultra-precision substrate surface with clear and straight steps could be obtained in both damaged regions and undamaged regions.

The experimental results showed good accordance with the crystal parameters, proving the practicability of simulating idealized step-and-terrace structures to approach the final steps morphologies. Idealized step-and-terrace structures of different miscuts and compositions were built and analyzed. Regarding the miscuts, it was found that the periodicity and the degree of order of the step structures were dependent on the miscut direction (step edge direction). For some specific miscut direction, the step structure was more regular with lower periodicity. Regarding the compositions, five possible compositions (i.e., Al~Al-O step, Al-O step, Al~Al step, Al step and O step) were found through idealized step structures because of the removal selectivity and the Al-O-Al trilayer structure. Five compositions shared the same periodicity, while the regulation could be ranked as: Al step ≈ O step > Al~Al step > Al-O step > Al~Al-O step. Furthermore, many subtle atomic structures (e.g., steps with different sawteeth, as well as steps with straight and zigzag edges) would form as the composition and the miscut direction changed.

To better understand the step formation, comparisons between experiments and simulations of different surface compositions were conducted. The structure on the polished surface was in better accordance with the model of single-atom steps (Al steps or O steps), whereas small regions of steps were in rough agreement with the step models of other compositions, indicating selectivity of different surface atom groups under different conditions during the polishing procedure. As for the regions without damage, the morphology was rather flat, so that the chemical reaction would play the leading role. The weaker chemical bonds, such as the bonds between Al-O-Al trilayers, would be broken first, so the morphology would change gradually from no steps to blur steps and to clear steps. This kind of formation would be prone to maintain the state of low energy. The single-atom steps had the lowest surface energy. Hence, it was natural to find good accordance between the experimental steps and the simulation model of single-atom steps. As for the regions with damage, some steps appeared at the peaks first. It means that before all the damage being removed the steps had already been formed. Because the energy of the peaks on the wafer was higher, that small scale of steps manifested on the peaks by chemical reaction first. In the same time, the mechanical action took on larger areas, the morphology changed a lot, from big waves of steps to small waves of steps and finally to straight steps. And because of the non-uniformity energy distribution, there was something higher than the steps on the step edges, which may be the unremoved hydration layer. Because the area of the regions with damage would be smaller and smaller with the polishing going on, most of the steps would also accord well with the model of single-atom steps. However, other step models also showed their appearance occasionally.

In addition to the mechanism of the step, the results also offered a tentative idea to design microscopic atomic step structures through crystal analysis by the macroscopic method of polishing. The simulation showed that step structures changed as the miscut and the composition changed. Different step structures, even more subtle atomic structures could be constructed by changing these two factors. Since some of the models accorded well with the steps of the experiments, it is possible to design step structures in this way by further studies on the experimental conditions.

## Conclusion

The present work may help to explain the mechanism of the step formation during the polishing process. Differences in step evolutions were studied between the regions without damage and the regions with damage by AFM experiments. The morphology of the damage-free region changed in line with ‘no steps - blur steps - clear steps’ chronologically. Whereas, the morphology of the damaged region changed as ‘small waves of steps - big waves of steps - straight steps’. Besides, different step models of different miscuts and compositions (i.e., Al~Al-O step, Al-O step, Al~Al step, Al step and O step) were simulated, finding that the single-atom steps (Al steps or O steps) with the miscut direction of a-axis were more regular. It was also found that more subtle atomic structures(e.g., steps with different sawteeth, and steps with zigzag edges) varied as the miscuts and compositions changed. Moreover, a comparison between the experiment and the simulation was performed. In general, the steps of the polished surface accorded well with the model of single-atom steps. And in some small regions, the steps of the polished surface agreed roughly with other step models.

## Methods

### Experimental Methods

Standard 2-inch c (0001) wafers of single crystal sapphire (Yunnan Sapphire Technology, China) were used in the experiments. Polishing experiments were conducted through CETR CP-4 machine (Bruker Nano Surfaces, Germany). Silica slurry with a pH value of 10.78 and the polyurethane polishing pad (Shenzhen Fangda Grinding Technology, China) were used. The initial roughness of the wafers was about 15 nm (measured with the MicroXAM-3D optical microscope, Aubat Precision Industry, China). To observe the evolution of step morphologies, the wafer surface was measured by AFM (Nanoman VS, Veeco, USA) every 20 minutes during polishing (with a pressure of 3.3 psi, a slurry supplying rate of 70 ml/min, an upper plating rotating speed of 120 r/min). To improve the clarity of the explanation, the region without damage was defined as the region of about 2 *mm*^2^ that had no scratches and other damage at the beginning stage of the polishing procedure. And the region with damage was defined as the region of about 2 *mm*^2^ that had damage at the beginning. Five points of regions without damage and five points of regions with damage were marked on the wafer. For further HRTEM (FEI Titan 80–300 Cs corrector TEM, USA) observations, a cross-sectional sample perpendicular to the step edges was made by Focused Ion Beam (FEI Quanta 200 FEG, USA) after polishing. The sapphire surface was coated with a noncrystalline carbon layer of about 5 nm thickness to make sure of the electric conduction and a noncrystalline platinum layer of about 300 nm thickness to protect the sapphire surface. The thickness of the TEM sample was controlled lower than 50 nm.

### Theoretical Models

**Figure 7:**
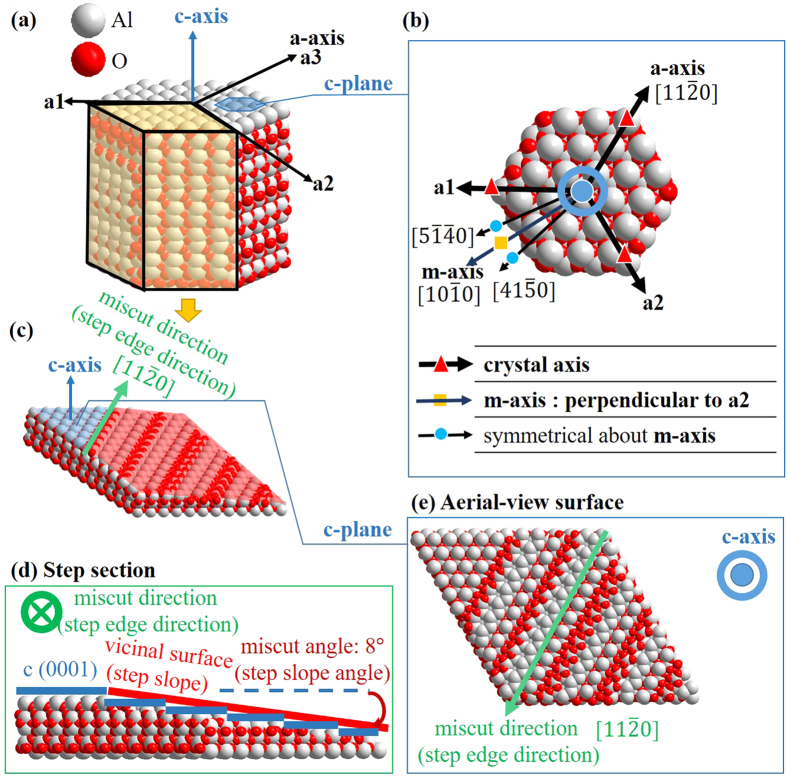
(**a**) Bulk crystal of the sapphire. (**b**) Four axes (a-axis: 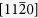, m-axis: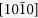, 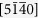, 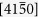) were selected as the miscut directions (step edge directions), respectively. 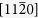 is the crystal axis of the sapphire. 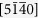 and 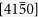 are symmetrical about 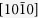. (**c**) The idealized step structure with the step edge along a-axis and the miscut angle (step slope angle) of 8° was shown as an example. (**d**) Step section: image observed upon the step edge. (**e**) Aerial-view surface: image observed upon the c-axis.

To better understand the step morphologies, idealized step structures of different miscuts and different compositions were constructed. As shown in Fig. 7(a,b), two special axes: a-axis 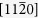, m-axis 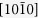, and two ordinary axes: 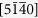, 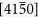, were selected as the miscut directions (step edge directions), respectively. Among them, a-axis is the crystal axes of sapphire. Moreover, 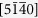 and 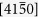 are symmetrical about m-axis. Since the step structure manifested on the surface, the surface would not be a perfectly c (0001) surface. The wafer surface was more likely a vicinal (0001) surface (step slope) formed with an ultra-small miscut angle along a miscut direction. The miscut angle was defined as the angle between the vicinal (0001) surface and the (0001) surface. The step structure model with the edge along a-axis and the miscut angle (step slope angle) of 8° is shown as an example in Fig. 7(c). The image looked down on the miscut direction is termed the step section [shown in Fig. 7(d)]. The surface looked along the c-axis is termed the aerial-view surface [shown in Fig. 7(e)].

**Figure 8:**
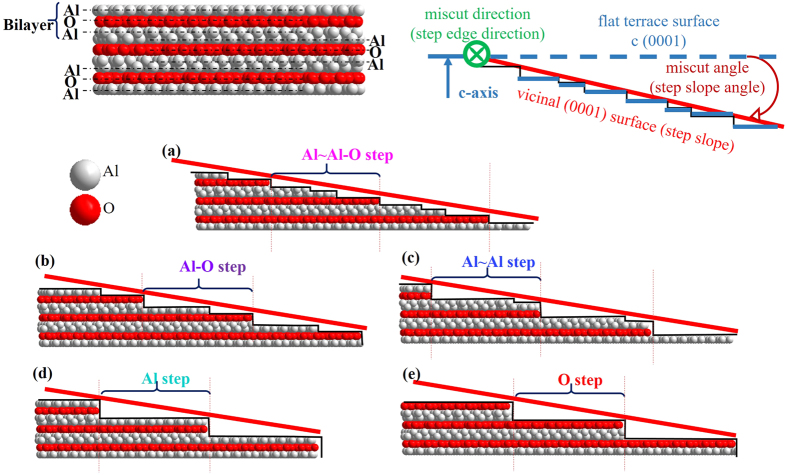
Five kinds of step compositions. (**a**) Al~Al-O step: An Al~Al-O step contains two Al layers and one O layer. (**b**) Al-O step. (**c**) Al~Al step: An Al~Al step contains two Al layers. (**d**) Al step: The flat terrace surface c (0001) is terminated with Al. (**e**) O step: The flat terrace surface c (0001) is terminated with O.

The sapphire crystal is the layer stacking structure (Al-O-Al~Al-O-Al~Al-O-Al…). The structure of one trilayer is Al-O-Al (shown in Fig. 8). Hence, the initial step model shown in Fig. 7 is the Al~Al-O step, with the assumption that the atoms are removed equally. However, atoms are usually removed selectively. Due to the difference in the bond strength, the bonds between the trilayers (for example Al~Al) may be broken first, because they are weaker than the bonds within the trilayers (for example Al-O). Also, due to the difference in binding energy, different surface atom groups of the surface would be combined and taken off selectively, causing the overall result of different terminations, whose existence had been proven in other research^44^. If the surface is terminated with O atoms, the step would naturally be the O step. According to these selectivities, there are five possible step compositions: Al~Al-O step, Al-O step, Al~Al step, Al step, O step (shown in Fig. 8). The step herein means the minimal repeating unit (in spite of the distinctions of the trilayers). As shown in Fig. 8, for the Al-O step and the single-atom step (Al step or O step), one step is within one trilayer, the total height of one step is the height of the trilayer h (0.216 nm). For the Al~Al-O step and the Al~Al step, one step is within two trilayers, while the total height of one step is also the height of the trilayer (see Section S1). Therefore, only statistics of the step widths were conducted to study the step structure.

After analyzing the influences of different miscuts and compositions with idealized models, a comparison between models and experiments was done for deep insight into the step formation mechanism during polishing. In that part, the miscut of the step structure model was restricted to the real miscut of the sapphire wafer used in the experiment, with the miscut direction (step edge direction) and the miscut angle (step slope angle) calculated from the experimental measurements.

## Additional Information

**How to cite this article**: Wang, R. *et al*. Atomic Step Formation on Sapphire Surface in Ultra-precision Manufacturing. *Sci. Rep.* **6**, 29964; doi: 10.1038/srep29964 (2016).

## Supplementary Material

Supplementary Information
