# Supplementary material for: Atomic Step Formation on Sapphire Surface in Ultra-precision Manufacturing
Source: Sci Rep. 2016 Jul 22;6:29964. doi: 10.1038/srep29964 (PMC4957212; doi:10.1038/srep29964)
Supplement: Supplementary Information [file srep29964-s1.pdf]

Supplementary Information

Atomic Step Formation on Sapphire Surface in Ultra-precision Manufacturing

Wang Rongrong[a], Guo Dan[a,*], Xie Guoxin [a,*], Pan Guoshun [a,b]

[a] *State Key Laboratory of Tribology, Tsinghua University, Beijing 100084, China*
[b] *Guangdong Provincial Key Laboratory of Optomechatronics, Shenzhen 518057, China*

[*] Corresponding author
Email address: guodan26@mail.tsinghua.edu.cn (Guo Dan), Xie-gx@163.com (Xie Guoxin)

S1. Step sections of different compositions.

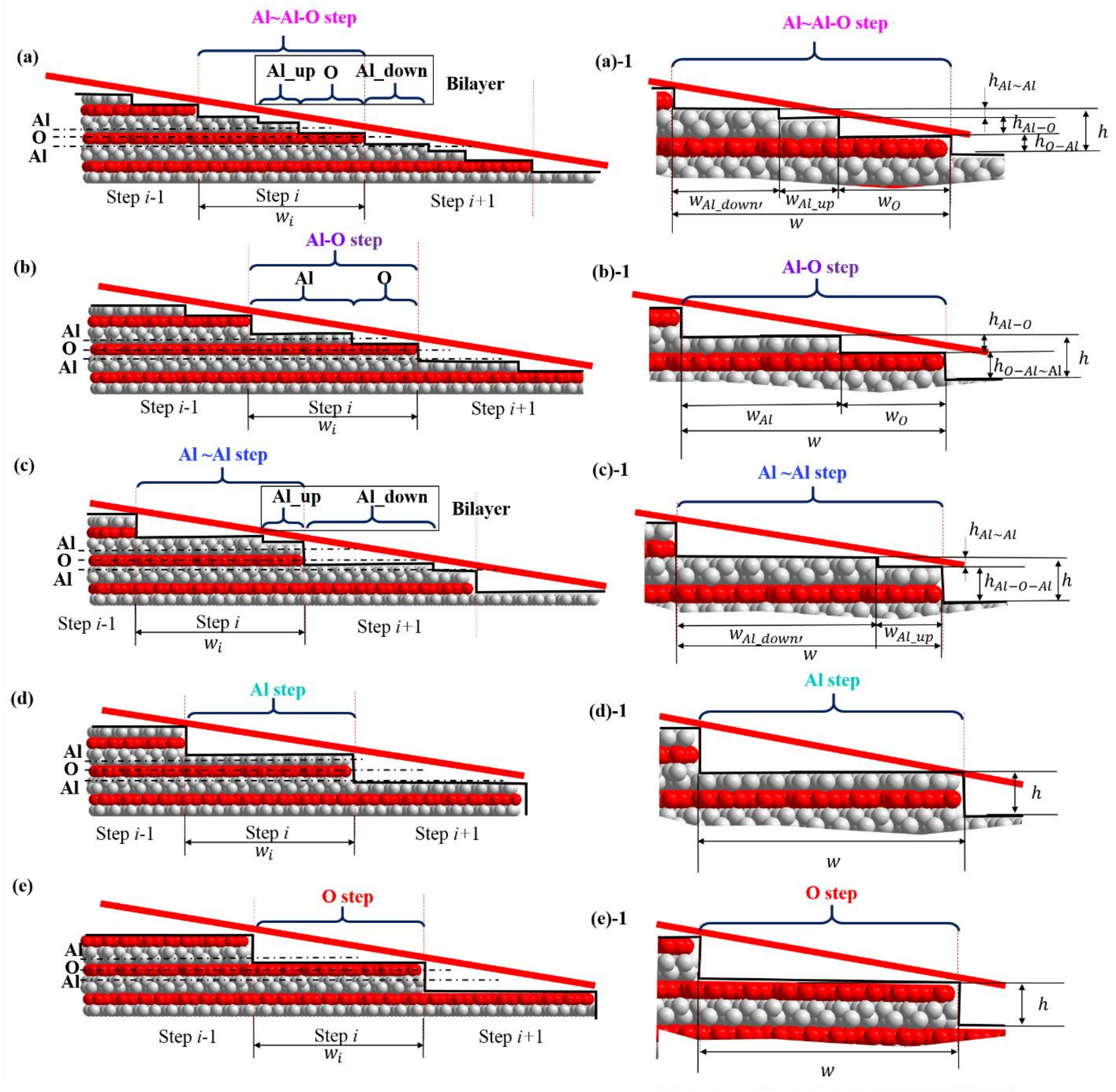

Figure S1. Step sections of different compositions. (a) Al~Al-O step. (b) Al-O step. (c) Al~Al step. (d) Al step. (e) O step.

S1.1 Step heights of different compositions.

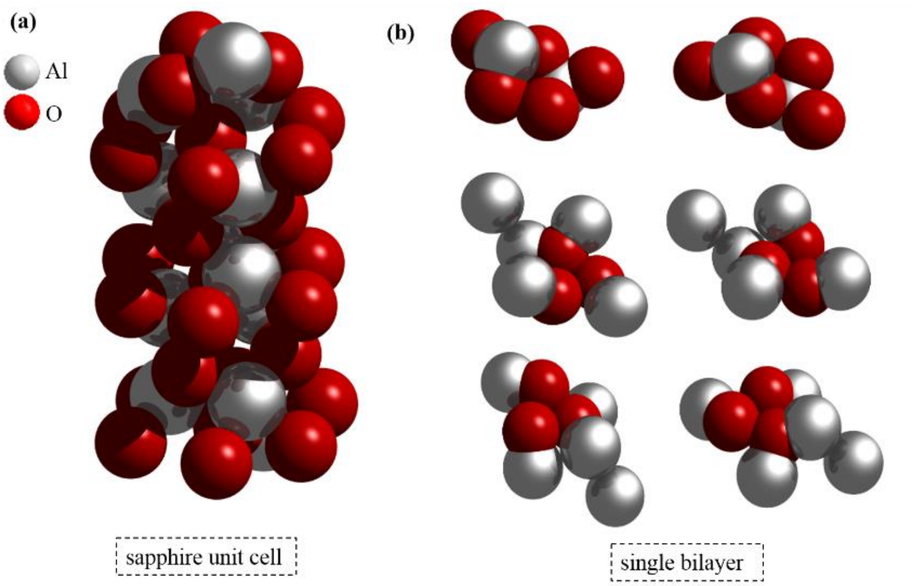

Figure S2. (a) Sapphire unit cell. (b) 6 kinds of single trilayers.

Figure S1 shows step sections of different compositions detailed by one step on an enlarged scale. As is shown in Figure S2, there are 6 kinds of single trilayers in one sapphire unit cell and every two trilayers are chirally symmetrical. The height of one trilayer $\boldsymbol{h}$ is 0.216 nm, consisting of the height of itself $\boldsymbol{h_{Al-O-Al}}$ and the height between the adjacent trilayers $\boldsymbol{h_{Al\sim Al}}$. As is shown in Figure S1, one Al-O step or one single atom step (Al step or O step) is within one trilayer, so the height of one step is the trilayer height $\boldsymbol{h}$ (0.216 nm). However, one Al~Al-O step or one Al~Al step is within two trilayers, because the height gaps within the step should be smaller than the height gap between the steps. One Al~Al-O step consists of the Al_down layer of the previous trilayer and the Al_up and the O layer of the current trilayer. One Al~Al step consists of the Al_down layer of the previous trilayer and the Al_up layer of the current trilayer, while the total height of one Al~Al-O step or Al~Al step is also the trilayer height $\boldsymbol{h}$ (0.216 nm), which can be seen in Figure S1(a)-1 and Figure S1(c)-1. For a simple geometric relationship, the step width will be $\boldsymbol{w} = \boldsymbol{h} \cdot \cot \boldsymbol{\theta}$ ($\boldsymbol{\theta}$ is the miscut angle). While due to the presence of atomic

distance, the step widths would be not perfectly equal to the geometric value. Therefore, steps were sequentially numbered as i downwards the c-axis to do the statistics of the step widths for further study of the step structure. Accordingly, $\boldsymbol{w_i}$ was defined as the width of the step $\boldsymbol{i}$.

S1.2 Step structures of different compositions.

As is shown in Figure S1, the Al~Al-O step has 3 layers, the Al-O and Al~Al step has 2 layers and the height between adjacent Al layers is smaller than that of adjacent Al and O layers. Hence, if the compositions are sorted from regular step structures to less regular ones, the ranking will be: Al step ≈ O step > Al~Al step > Al-O step > Al~Al-O step.

S2. Aerial-view surfaces of different miscuts.

In the main text, the general influences of different miscuts and compositions were discussed. Actually, the miscuts and compositions would also influence the more subtle arrangements of step structures.

For different compositions, subtle atomic arrangements of the step structures have been shown in Figure S1. It can be seen that the Al~Al-O step has two Al layers and one O layer, the Al-O step has one Al layer and one O layer, the Al~Al step has two Al layers, and the Al flat terrace surface is terminated with Al and the O flat terrace surface is terminated with O.

As for the influences of the miscut on the subtle atomic arrangements, aerial-view surfaces of different miscut directions (step edge directions) are shown in Figure S3 when the miscut angle (step slope angle) was 5°. The step edge of the a-axis is straight, and the step edge atoms arrange

closely. The step edges of the m-axis, $[5\bar{1}\bar{4}0]$, and $[41\bar{5}0]$ are zigzag. Among them, the step edge of the m-axis is with a sawtooth shape of an isosceles triangle, and the step edge of the $[5\bar{1}\bar{4}0]$ share the same shape to the step edge of $[41\bar{5}0]$.

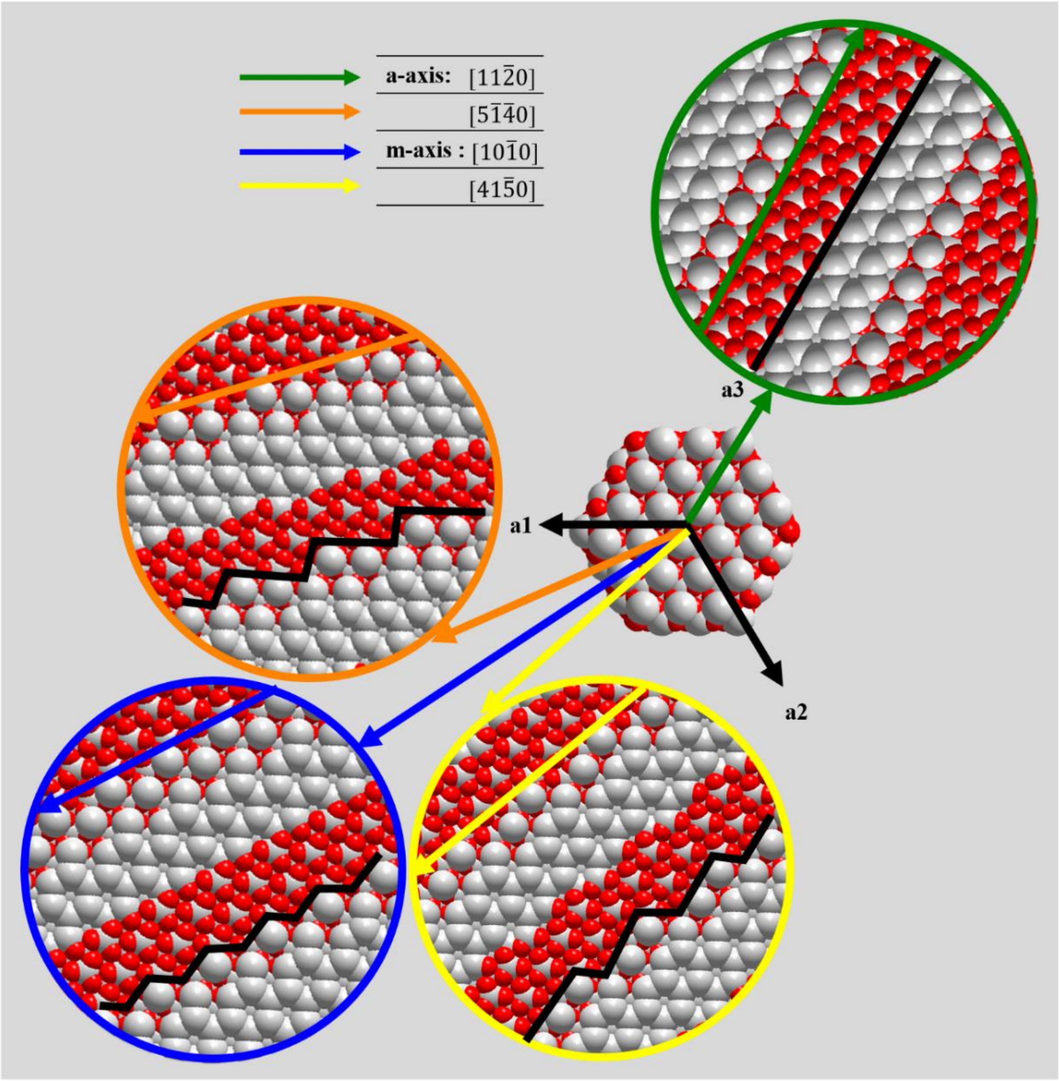

Figure S3. Aerial-view surfaces of different miscut directions (step edge directions) when the miscut angle was 5°.

S3. Fluctuation of the step parameters.

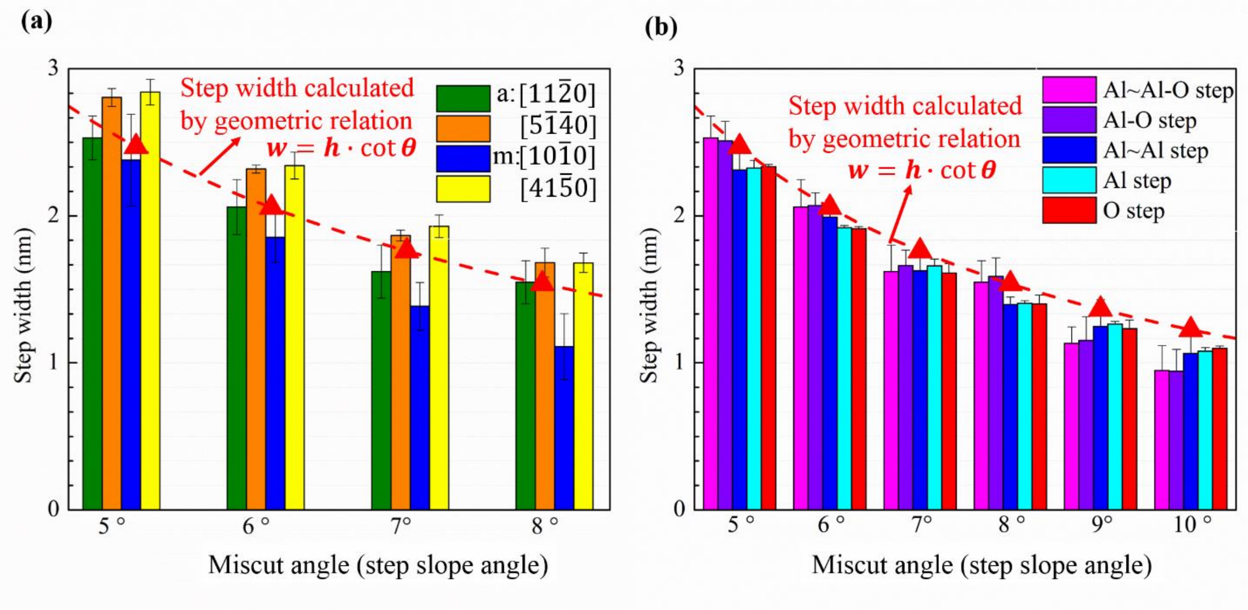

Figure S4. (a) Al step widths as a function of miscut angles (step slope angles) at different miscut directions (step edge directions). (b) Step widths as a function of miscut angles (step slope angles) for different compositions.

To have a better understanding of the step parameter of different miscuts, the mean values of 24 Al step widths for different miscut directions (step edge directions) and various miscut angles (step slope angles) were calculated and summarized in Figure S4(a). The step widths of the a-axis accorded better with the geometric calculations (with a difference of about 0.2 nm). The step width of the m-axis approached the curve of geometric relation as the miscut angle decreased (with the

difference changing from 0.4 nm to 0.1 nm). The step widths of the $[5\bar{1}\bar{4}0]$ coincided with those of the $[41\bar{5}0]$, which was probably due to the fact that these two directions were symmetrical about the m-axis. All in all, if the miscut directions (step edge directions) were sorted from bigger fluctuation step parameters to smaller ones, the ranking would be: $[11\bar{2}0]$ (a-axis) > $[10\bar{1}0]$ (m-axis) > $[5\bar{1}\bar{4}0] \approx [41\bar{5}0]$.

Means and standard deviations of 24 step widths were calculated and summarized in Figure S4(b) for different compositions with the change of miscut angle (step slope angle). Generally, the widths of Al steps, O steps and Al~Al steps roughly coincided with each other (with about 0.01 nm difference), and they accorded better with the geometric calculations (with a nearly constant difference 0.15 nm). On the contrary, the widths of Al~Al-O steps and Al-O steps almost coincided with each other (with about 0.01 nm difference), and they roughly approached the geometric relation line as the miscut angle decreased (with the difference changing from 0.3 nm to 0.05 nm). All in all, if the compositions are sorted from bigger fluctuation step parameters to smaller ones, the ranking will be: Al step ≈ O step ≈ Al~Al step > Al-O step ≈ Al~Al-O step.

S4. Calculations of the miscut parameters.

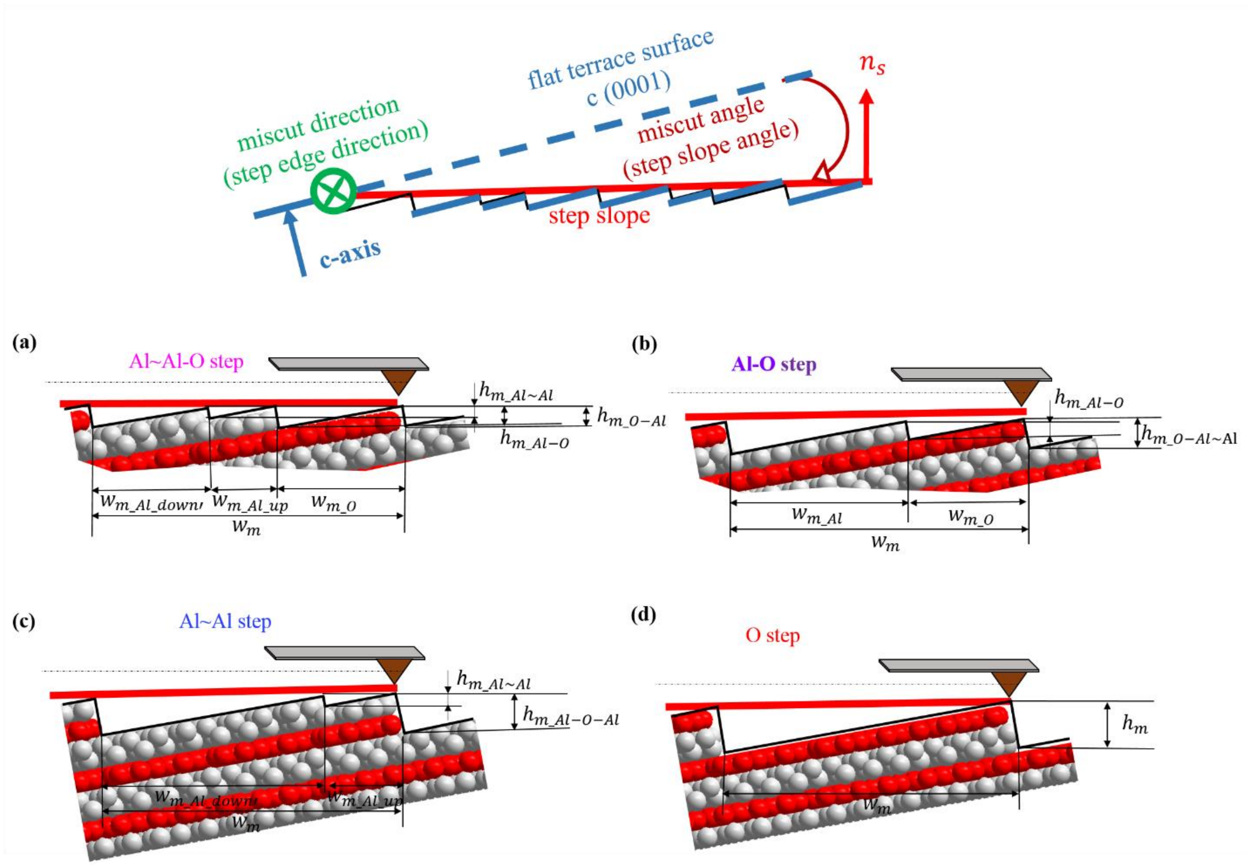

Figure S5. Step parameters with the horizontal axis along the vicinal (0001) surface (step slope) and the vertical axis along n_s (which is normal to step slope). (a) Al~Al-O step. (b) Al-O step. (c) Al~Al step. (d) O step.

In the AFM experiment the vertical axis is along n_s (which is normal to step slope), thus the step sections in Figure S5 were rotated to make the horizontal axis along the vicinal (0001) surface (step slope) and the vertical axis along n_s to analyze the step parameters. As shown in Figure S5(a), the width of Al~Al-O step along the vicinal (0001) surface (step slope) is $w_m = w_{m\_Al\_down'} + w_{m\_Al\_up} + w_{m\_O} = w_{Al\_down'}/\cos\theta + w_{Al\_up}/\cos\theta + w_O/\cos\theta = w/\cos\theta$. Similarly, the step width of other compositions along the vicinal (0001) surface (step slope) is also $w_m = w/\cos\theta$. As the height means the gap between the highest point and the lowest point of one

step, it does not have the additivity which is shown above in the step width. For Al~Al-O step, the step height along n_s is $h_m = \max\{h_{m\_Al\sim Al}, h_{m\_Al-O}, h_{m\_O-Al}\} = h_{m\_Al-O} = h_{Al-O} \cdot \cos\theta$. Similarly, step heights along n_s of different compositions are shown in Table S1.

The miscut angle θ of the wafer used in the experiment was lower than 0.5°, thus $\cos\theta$ was approximately equal to 1 and the width along the vicinal (0001) surface (step slope) was approximately equal to the width along the flat terrace surface (approximated values of the heights are shown in Table S1).

Table S1. Step heights along n_s of different compositions.

	Al~Al-O step	Al-O step	Al~Al step	Single atom step (Al step or O step)
h_m (Height along n_s)	$h_{Al-O} \cdot \cos\theta.$	$h_{O-Al\sim Al} \cdot \cos\theta$	$h_{Al-O-Al} \cdot \cos\theta$	$h \cdot \cos\theta$
Approximated value	h_{Al-O} (0.084 nm)	$h_{O-Al\sim Al}$ (0.133 nm)	$h_{Al-O-Al}$ (0.167 nm)	h (0.216 nm)

From the AFM sectional analysis in Figure 4 (d), the average step heights along n_s are about 0.2 nm. The step heights are quite close to the single trilayer height (0.216 nm), indicating the

structural consistency between the experimentally obtained steps and the idealized crystal steps. The average step widths along vicinal (0001) surface (step slope) were about 47.6 nm. Bring the step parameters with the horizontal axis along the vicinal (0001) surface (step slope) to the geometric relationship: $\boldsymbol{w} = \boldsymbol{h} \cdot \cot \boldsymbol{\theta}$, and it can be calculated that the $w_m = 2h_m/\sin 2\theta$. Thus the miscut angle (step slope angle) can be estimated to be around 0.26°. From the nominal miscut direction of the sapphire wafers used in the experiments and the ensurement of the calibration of the HRTEM zone axis [in Figure 5 (b)], the miscut direction (step edge direction) was a-axis.

S5. AFM images with the marked line.

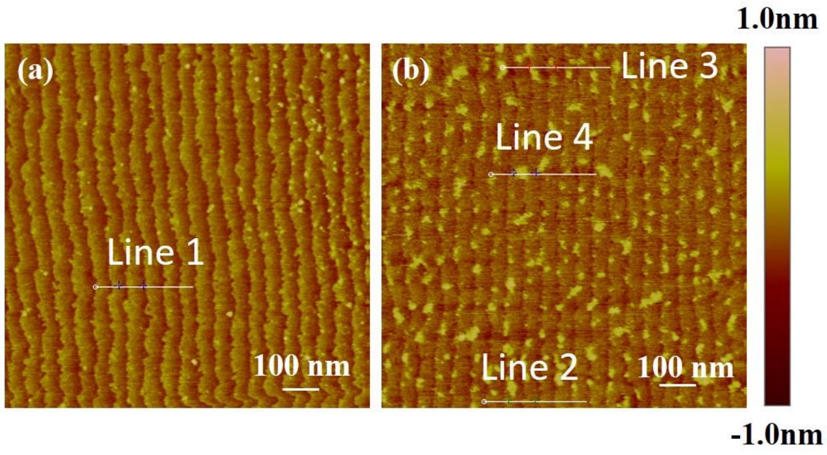

Figure S5. The experimental profile of Figure 6(a) was extracted from the Line 1. The experimental profile of Figure 6(b) was extracted from the Line 2. The experimental profile of Figure 6(c) was extracted from the Line 3. The experimental profile of Figure 6(d) was extracted from the Line 4.
